# Comparison of Hemodynamics After Fenestrated, Branched, and Chimney Endovascular Aneurysm Repair Employing Computational Fluid Dynamics

**DOI:** 10.3390/jcm15051914

**Published:** 2026-03-03

**Authors:** Stavros Malatos, Spyridon Katsoudas, Anastasios Raptis, Laura Fazzini, Petroula Nana, George Kouvelos, Athanasios Giannoukas, Michalis Xenos, Miltiadis Matsagkas

**Affiliations:** 1 Department of Vascular Surgery, Faculty of Medicine, School of Health Sciences, University of Thessaly, 41334 Larissa, Greece; stavros.malatos@gmail.com (S.M.); geokouv@gmail.com (G.K.); giannouk@med.uth.gr (A.G.); 2 Department of Mathematics, University of Ioannina, 45110 Ioannina, Greece; s.katsoudas@uoi.gr (S.K.); mxenos@uoi.gr (M.X.); 3 Laboratory of Biofluid Mechanics & Biomedical Technology, School of Mechanical Engineering, National Technical University of Athens, 15772 Zografou, Greece; raptistasos@mail.ntua.gr; 4 Clinical Engineer Department, Asl Roma 6, 00041 Ariccia, Italy; laurafazzini6@gmail.com; 5 German Aortic Center, Department of Vascular Medicine, University Hospital Eppendorf, 20251 Hamburg, Germany; petr.nana7@hotmail.com

**Keywords:** FEVAR, BEVAR, chEVAR, computational fluid dynamics, hemodynamic performance, endograft-specific flow analysis

## Abstract

**Background/Objectives:** This study compared the hemodynamic performance of fenestrated (FEVAR), branched (BEVAR), and chimney endovascular aortic aneurysm repair (chEVAR) in patients with complex aortic aneurysms. **Methods:** The pre- (native) and post-endovascular repair (endograft-defined) blood lumen was reconstructed from computed tomography angiographies of nine (9) elective patients treated with FEVAR (*n* = 3), BEVAR (*n* = 3), and chEVAR (*n* = 3). Computational fluid dynamics (CFD) simulations obtained blood flow properties. Velocity magnitude, wall shear stress (WSS), time-averaged wall shear stress (TAWSS), oscillatory shear index (OSI), relative residence time (RRT), and local normalized helicity (LNH) were computed at peak systole and mid-diastole. The hemodynamic data were statistically analyzed to evaluate correlations between FEVAR, BEVAR, and chEVAR, focusing on targeted visceral arteries. **Results:** Only slight differences were observed regarding RRT, OSI, and TAWSS between FEVAR and BEVAR, whereas the chEVAR group demonstrated a marked deviation from both. In FEVAR, the postoperative helical flow structures appeared more compact, while in BEVAR they were more developed and exhibited a more rotational configuration. The LNH of the visceral vessel patterns exhibited similar qualitative features across groups. Regarding TAWSS, higher values were found in BEVAR, whereas chEVAR showed the lowest. **Conclusions:** FEVAR, BEVAR, and chEVAR improved postoperative blood flow characteristics toward near-physiological conditions, reducing undesired flow patterns and recirculation zones. FEVAR showed more stable visceral flow, and BEVAR demonstrated higher flow rates and fewer recirculation zones, while chEVAR exhibited more streamlined visceral artery flow with reduced regurgitation at bridging stent entries. Despite variations, all approaches effectively preserved visceral artery perfusion.

## 1. Introduction

Endovascular repair using fenestrated and branched endografts is the first-line treatment for patients with juxtarenal and pararenal abdominal aortic aneurysms (AAAs) [1,2], while off-the-shelf solutions may may be used in urgent settings or when the anatomy does not permit a custom-made endograft application [3]. Endovascular treatment for complex aneurysms has emerged as a preferred approach in contemporary medical practice. The advancements in endovascular solutions, including the use of custom-made grafts that incorporate branches and fenestrations, alongside off-the-shelf devices, have greatly enhanced the feasibility and effectiveness of these interventions. Despite the high technical success and the low early mortality and morbidity recorded in large retrospective cohorts of high-volume aortic centers, the durability of complex endovascular approaches is still questioned due to the higher reintervention rates [4,5,6]. Especially for complex endovascular aortic repair, target vessel adverse events are the leading cause for reintervention during the mid-term follow-up [5,6]. Previous analyses on fenestrated versus branched devices have shown that fenestrations, when applicable, are superior to branches in terms of target vessel instability [7].

Recent studies show that postoperative hemodynamics in the visceral branches critically influence F/BEVAR and chEVAR success [8,9]. Abnormal flow patterns and shear-stress distributions have been linked to branch stenosis, impaired organ perfusion, and increase target vessel instability events. Given the variability in endograft techniques, understanding the impact of configuration on target vessels’ blood flow remains an important unresolved issue [1,10,11]. Due to the lack of patient-specific hemodynamic data at the inlet and outlets of the 3D vascular models, advanced multiscale cardiovascular models, 3D–1D, can generate the required waveforms for the 3D simulations and assist with understanding complex hemodynamic features [12].

Over the past two decades, image-based computational hemodynamics has progressed through advances in multiscale and fluid–structure interaction (FSI) modeling of large arteries. Key developments include physiologically consistent outflow boundary conditions for patient-specific simulations and fully coupled multiscale–FSI frameworks capturing arterial wall deformation under pulsatile flow [13,14]. These methodologies have been applied to endovascular repair, enabling detailed assessment of near-wall hemodynamics and shear-stress-related indices that influence branch durability after complex EVAR [11].

This study aims to compare the blood flow characteristics in target vessels after complex endovascular aortic aneurysm repair using different approaches (FEVAR, BEVAR, and chEVAR) using patient-specific computational models.

## 2. Materials and Methods

### 2.1. Study Design and Patient Selection

**Figure A1:**
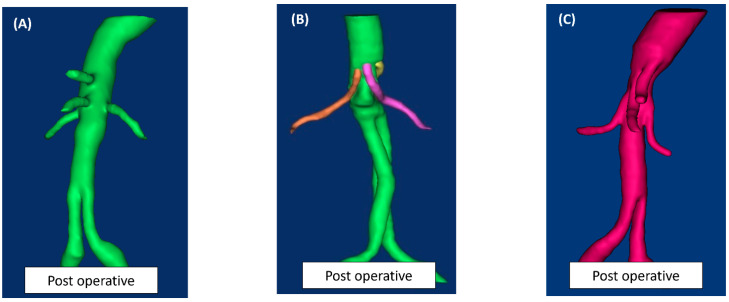
Reconstructions of postoperative cases: (**A**) FEVAR, (**B**) chEVAR, and (**C**) BEVAR.

A retrospective analysis was conducted on blood flow features pre- and post-FEVAR, BEVAR, and chEVAR (see Figure A1) among patients that were managed for degenerative complex aortic aneurysms between 2018 and 2021 in a single-tertiary center. Computed tomography angiography (CTA) scans were used to define the pre- and post-repair blood flow lumens of patients with complex aortic aneurysms using computational fluid dynamics simulations, with a special focus on the hemodynamic properties of the visceral vessels [8,12].

Patients with juxtarenal, pararenal, and thoracoabdominal aortic aneurysms that were managed using FEVAR, BEVAR, or chEVAR under an elective setting and completed the one-month CTA (slice thickness < 1 mm and arterial phase) imaging follow-up were considered eligible. A revascularization and bridging of three (for chEVAR) or four target vessels was a criterion for inclusion. The decision for the treatment approach relied on patient-specific characteristics, anatomy of the aorta and target vessels, and availability of devices and materials. Patients managed for aortic dissections (acute or chronic), pseudoaneurysms, or who had an anamnesis of previous endovascular or open aortic aneurysm repair were excluded. No surgeon-modified endografts were used during the study period.

Pseudonymized pre- and postoperative computed tomography angiographies (CTA) of 3 cases for each technique (FEVAR, BEVAR, and chEVAR) were collected and analyzed. This study complied with the declaration of Helsinki. No ethics committee approval was required due to its retrospective nature and pseudonymized information.

### 2.2. Endovascular Approaches

FEVAR was used for the treatment of juxta- and pararenal aortic aneurysms, if the apposition of the device on the aortic wall and especially the level of the target vessels was expected. All FEVAR cases were managed with company-manufactured custom-made devices relying on the Zenith Platform (Cook Medical, Bloomington, IN, USA) [15]. BEVAR was chosen for patients with more extensive aortic pathologies, including thoracoabdominal aneurysms, when a device apposition on the aortic wall was not expected. Patients managed with BEVAR were treated using off-the-shelf branched devices (T-Branch device, William Cook Europe, Bjaeverskov, Denmark), all relying on the Zenith platform. ChEVAR was used in urgent cases (symptomatic or ruptured juxta- or pararenal aortic aneurysms) or for aneurysms with a diameter over 70 mm, when a custom-made device was not available due to the long-time interval for device design and manufacture. For patients managed with chEVAR, the Endurant IIs (Medtronic, Santa Rosa, CA, USA) device was used as the aortic component. All aortic devices were oversized by 20%, except for chEVAR, where the aortic device was oversized by 30%, respecting that three target vessels were revascularized [16].

All target vessels were bridged using the same bridging stents (BeGraft Peripheral, Bentley, Hechingen, Germany). The bridging stent diameter and length were decided-upon the technical features of the main device based on the distal landing zone and anatomy of the target vessels. Details on anatomic parameters and devices are presented in Table 1.

### 2.3. Modeling

The 3D model of the FEVAR, chEVAR, and BEVAR systems, including the celiac artery (CA), superior mesenteric artery (SMA), left renal artery (LRA), and right renal artery (RRA), was constructed using the CTA scan for each patient (Figure A1A). The reconstruction of the DICOM images into a 3D lumen model was performed using the methodology mentioned in previous studies [8,9,12].

The reconstruction of the geometry in question started just above the CA and ended just below the iliac bifurcation, while the region of interest was the visceral aorta and specifically the CA, SMA, RRA, and LRA. For the FEVAR and BEVAR cases, the segmentation was conducted as a single combined structure. For chEVAR cases, however, the lumens of the covered abdominal aorta and the stented visceral arteries (SMA, RRA, and LRA) were segmented individually using the same software (see Figure A1). The optimal smoothing factor value (SFV) was determined to be 0.05, based on a smoothing parameter study performed on renal and mesenteric diameters using the Vascular Modeling Toolkit (VMTK) (version 1.4.0) [8].

The following hemodynamic parameters were calculated: time-averaged wall shear stress (TAWSS), oscillatory shear index (OSI), relative residence time (RRT), and local normalized helicity (LNH). The postoperative results presented correspond to a representative patient from each group, as no significant differences between patients were observed.

**Figure A2:**
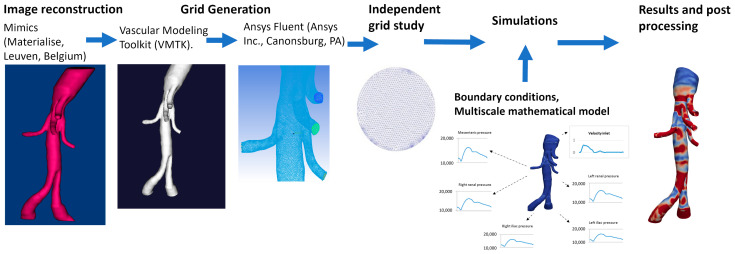
Flowchart of the methodology. Flow rate in boundary conditions is in ml/s and pressure in Pa.

Numerical simulations were performed with the software package Ansys Fluent 16.1 (Ansys Inc., Canonsburg, PA, USA). Blood was considered a Newtonian fluid with a density of *ρ* = 1050 kg/m^−3^ and a kinematic viscosity of *v* = 3.2 × 10^−6^ m^3^ s^−1^. The zero-velocity condition was applied on the surface of the endograft, following the rigid wall assumption. Over two million tetrahedral elements were discretized from the fluid domain, with an additional three-layer boundary and the inner element size being approximately 1 mm. The boundary conditions at the inlet and outlets were defined using the flow and pressure waveforms obtained from the multiscale model (0D-3D) developed in our previous studies [9,13]. Specifically, a flow waveform representing physiological (healthy) conditions, obtained from the literature, was applied to the input [9,12]. At the outlets, resistance-based boundary conditions (pressure waveforms) derived from the 1D mathematical model were imposed for each respective artery. The velocity profile at the outputs was almost parabolic during the systolic phase [8,12,17,18,19,20,21]. Convergence to the solution at each step was considered achieved when the computational error fell below 10^−4^. We exclusively used the results of the last cardiac cycle, avoiding any dynamic behavior of the first pulses [10]. Grid independence tests were performed in regions of predicted disturbed flow to determine the optimal mesh size. The simulation was considered grid-independent when the hemodynamic parameters (velocity and pressure) varied by less than 2% between successive mesh refinements [22]. In all 3D simulations, a parabolic velocity profile was prescribed at the inlet, a no-slip boundary condition was applied at the wall (i.e., along the stent-graft surface), and pressure waveforms were imposed at each outlet (see Figure A2). The outlet pressure waveforms were obtained from a one-dimensional hemodynamic model representing the complete arterial network [12,17,19,20,21,23,24,25,26]. Paraview version 5.13.3 (Kitware Inc., Clifton Park, NY, USA) software was used for the visualization of hemodynamic parameters.

### 2.4. Statistical Analysis

Differences in hemodynamic parameters (RRT, OSI, TAWSS) among the three endograft groups were assessed in the target vessels (SMA, LRA, RRA) using one-way ANOVA, with *p* < 0.05 considered statistically significant.

## 3. Results

### 3.1. Wall Shear Stress (WSS)

**Figure 1:**
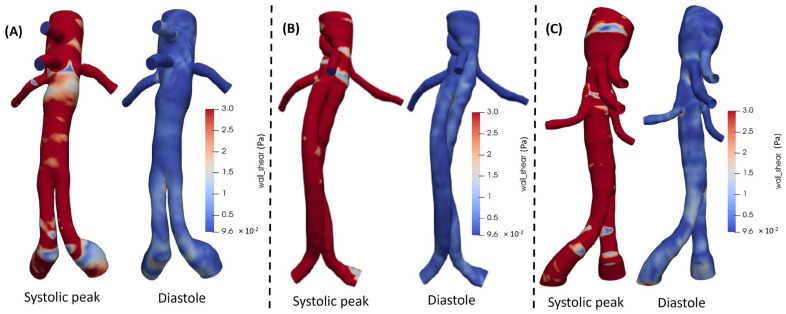
WSS in (**A**) FEVAR, (**B**) ChEVAR, and (**C**) BEVAR cases, for systolic peak and diastole.

Postoperatively, during the peak systole, WSS values showed a maximum value of 3 Pa for all designs (Figure 1). Higher WSS values across the whole structure and especially in the visceral arteries were observed for BEVAR cases, followed by intermediate values for FEVAR and the lowest values for chEVAR as shown in Table 2.

### 3.2. Local Normalized Helicity (LNH)

**Figure 2:**
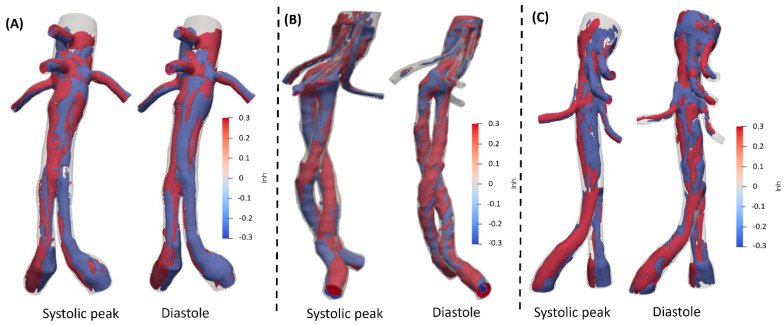
LNH in (**A**) FEVAR, (**B**) ChEVAR, and (**C**) BEVAR cases in different cardiac phases.

The helical structures in all three endograft groups are distributed evenly throughout the device (Figure 2). In FEVAR cases (Figure 2A), the helical patterns appear more compact and smoother compared to those observed in BEVAR (Figure 2C). Postoperatively, the helical structures in BEVAR were more pronounced and exhibited a clearer rotational configuration (|LNH| < 0.3). According to the literature, a threshold value of 0.3 is typically considered indicative of rotational flow structures [27,28,29]. In contrast, in FEVAR cases (Figure 2A), the local normalized helicity (LNH) shows less rotational behavior compared to chEVAR (Figure 2B). Additionally, in the visceral arteries of all groups, a relatively uniform distribution of helical structures was observed postoperatively.

### 3.3. Time-Averaged Wall Shear Stress (TAWSS)

**Figure 3:**
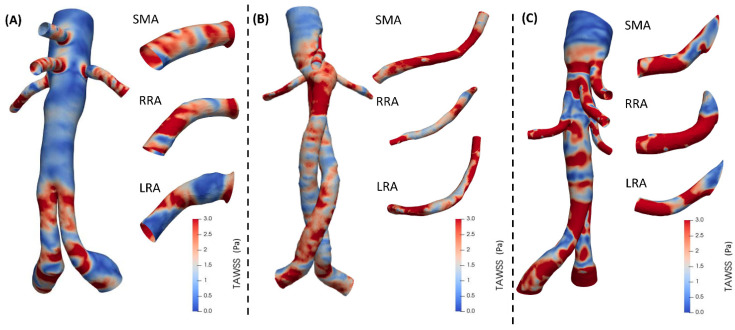
Time-averaged wall shear stress (TAWSS) in (**A**) FEVAR, (**B**) chEVAR, and (**C**) BEVAR cases.

The results were evaluated assuming a maximum TAWSS value of 3 Pa. As shown in Table 2, BEVAR exhibited the highest TAWSS values compared to the other two techniques. BEVAR and FEVAR demonstrated relatively high TAWSS, with only minor deviations between them, whereas chEVAR cases showed significantly lower TAWSS (Figure 3). In BEVAR cases, increased TAWSS was distributed across nearly the entire surface of the structure, while in FEVAR cases, moderate TAWSS values were observed primarily in the region below the renal arteries and above the iliac bifurcation (Figure 3A).

### 3.4. Oscillatory Shear Index (OSI) and Relative Residence Time (RRT)

**Figure A3:**
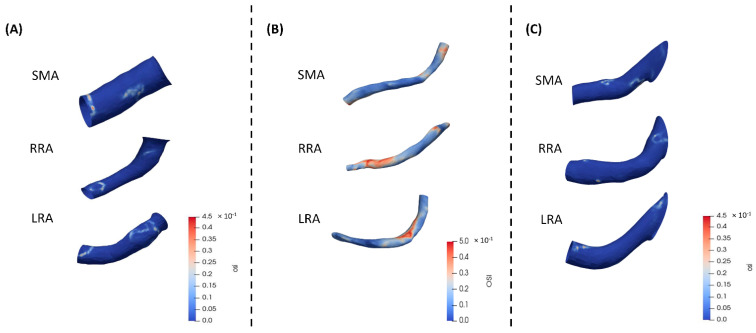
Oscillatory shear index (OSI) in (**A**) FEVAR, (**B**) chEVAR, and (**C**) ΒEVAR cases.

**Figure A4:**
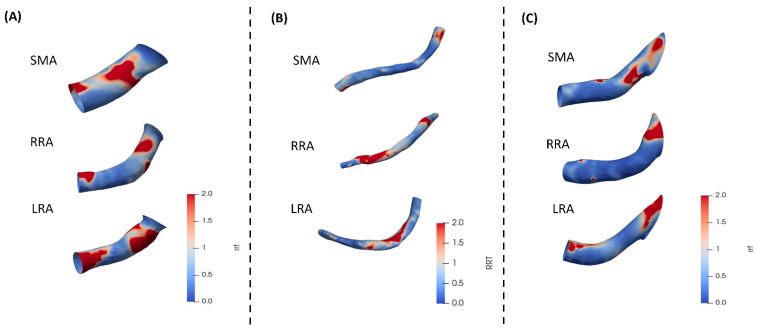
Relative residence time (RRT) in (**A**) FEVAR, (**B**) chEVAR, and (**C**) BEVAR cases.

There were areas of low OSI values in the main aortic endograft for all groups. More regions of low value of localized OSI mainly occurred in the FEVAR and BEVAR cases. OSI showed intense oscillations in individual locations of the visceral arteries in the chEVAR group. In postoperative BEVAR cases, the OSI showing increased values among the mesenteric and renal arteries (Figure A3C). In BEVAR target vessels, low levels of OSI in the proximal end and moderate values in the distal end were recorded, contrary to chEVAR, where a high-value localized OSI prevailed (Figure A3B,C). In all cases, the RRT values were mainly driven by the OSI patterns following the opposite behavior (Figure A3 and Figure A4).

### 3.5. Flow Comparison in FEVAR, chEVAR, and BEVAR

**Figure 4:**
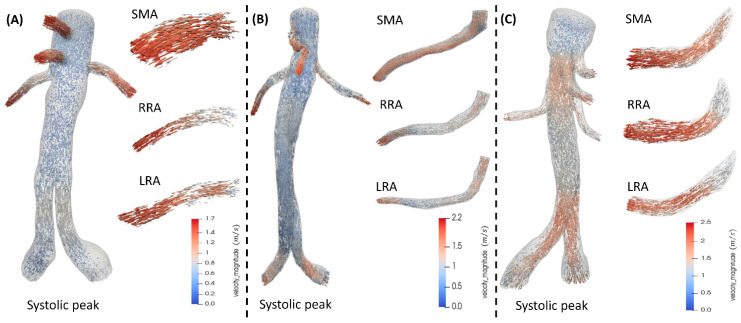
Flow velocity magnitude comparison in the secondary arteries at peak systole, the mesenteric artery (SMA), and the left and right renal arteries (LRA, RRA) for (**A**) FEVAR, (**B**) chEVAR, and (**C**) BEVAR cases.

The flow at peak systole in the FEVAR, chEVAR, and BEVAR groups is depicted in Figure 4. The maximum flow velocity in the visceral arteries was recorded in BEVAR ~2.5 m/s versus ~2.2 m/s in chEVAR and ~1.7 m/s in FEVAR cases (Table 2). No statistically significant differences in visceral vessel flow were observed between FEVAR and chEVAR. The endograft segment between the CA and renal arteries showed increased flow in BEVAR, while the flow was smoother in the main body of the other two techniques (Figure 4) than in chEVAR during the systolic phase.

### 3.6. Mean Visceral Hemodynamics

**Figure 5:**
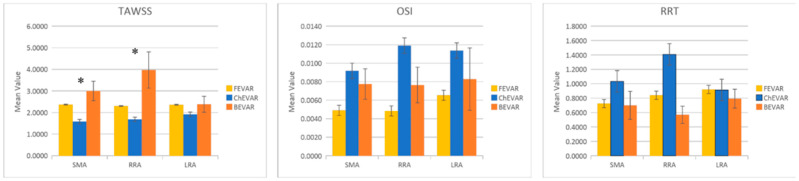
Mean values for the hemodynamic parameters (RRT, OSI, and TAWSS) between FEVAR, chEVAR, and BEVAR endograft groups, where * indicates statistically significant differences between groups.

No statistically significant differences were found in the RRT and OSI of the target vessels among the techniques. Comparing the mean values of hemodynamic parameters (RRT, OSI, and TAWSS), only slight differences were observed between FEVAR and BEVAR. In contrast, chEVAR showed deviation from the other two groups (Figure 5).

Statistically significant differences (*p* < 0.05) were observed for TAWSS for the SMA (*p* = 0.0162) and RRA (*p* = 0.0255) in the postoperative setting. Regurgitant flow in the visceral arteries was computed and compared across FEVAR, chEVAR, and BEVAR cases. chEVAR exhibited the highest mean value, FEVAR intermediate, and BEVAR the lowest (Table 3) [8,18].

## 4. Discussion

FEVAR/BEVAR expanded their targeted patient population and currently represent the recommended approach in patients with adequate anatomy and complex aortic aneurysms, while the chimney technique received a lot of criticism regarding the risk for failure of the proximal landing zone and higher endoleak rates and mainly represents a bailout approach [30,31,32]. FEVAR and chEVAR have been employed for the treatment of anatomically suitable complex aortic aneurysms. Regardless of the technique, endovascular management seems to provide a higher benefit in terms of mortality and morbidity perioperatively, while the need for reintervention still represents a major issue, with most of them though being minor and endovascularly performed without impact on survival [30,31,32]. Except for the main device, target vessels’ fate plays a significant role in FBEVAR and chEVAR clinical success, with endoleaks, occlusions, and instability affecting the durability of the procedure and being mainly related to branches [33]. In this study, a multiscale computational approach composed of simple holistic mathematical models combined with advanced 3D CFD simulations was used to provide clinicians with important information about patients’ hemodynamics. Clinicians can further assess the application of the three methods, determine the most appropriate treatment strategy for each patient with complex aneurysms, and anticipate potential postoperative complications. In this analysis evaluating the hemodynamic performance of fenestrated, branched devices and chimney approaches, it was shown that all techniques lead to a reconstruction of the aortic and visceral vessel geometry and hemodynamic results close to normal levels [1,8,10,11,12]. Higher-pressure values during the systolic phase were obtained proximally in the main device regarding target-EVAR cases followed by chEVAR, while the lowest values were recorded in FEVAR cases. Overall, FEVAR and BEVAR seem to provide similar smoother flow with less regurgitation than the chimney technique.

FEVAR and chEVAR are usually applied in anatomically similar complex abdominal aortic aneurysms, but their application may lead to distinct local hemodynamic changes. An overall improvement in hemodynamics was detected after repair with either technique, with improved hemodynamics towards normal values and reduced recirculation zones in the main graft and target vessels. Preoperatively, a disrupted and pro-thrombotic WSS pattern was observed in multiple regions of the aneurysm sac in both FEVAR and chEVAR cases. LNH analysis demonstrated improved organization of helical flow structures in the postoperative setting with both techniques, suggesting reduced thrombus formation. Likewise, TAWSS increased and OSI decreased after intervention, indicating a more stable and less disturbed flow environment. RRT was also locally diminished. Flow within the target vessels appeared more streamlined in chEVAR, whereas noticeable recirculation zones were present at the renal and superior mesenteric artery fenestrations (*p* = 0.06). This difference is likely attributable to the bridging stent configuration, as chimney grafts are oriented vertically, while fenestration bridging stents are positioned transversely. However, FEVAR showed less intense flow regurgitation in bridging stents.

Regarding the comparative findings on BEVAR and FEVAR, the hemodynamic characteristics after the repair were clearly improved regardless of the approach. The WSS profiles of BEVAR show slightly higher values compared to FEVAR. LNH in both cases improved by suppressing the disturbed blood flow but providing a more balanced distribution in FEVAR cases. The distribution of TAWSS in BEVAR was prominently increased in the entire structure, which leads to low values of OSI and RRT. The flow in target vessels seems to be more streamlined in FEVAR but presents an instant increase compared to BEVAR, where the flow develops gradually, with BEVAR showing slightly less recirculation zones in the branches in contrast to FEVAR, where the flow is more stable. These findings justify the fact that in FEVAR, we have less occlusion and more stable technical outcomes in target vessels, as also reported in the literature [34,35].

### Limitations

The elastic behavior of the graft material was not considered, and the luminal surface was assumed to be rigid. Blood was treated as a single continuum medium, although it could alternatively be modeled as a non-Newtonian fluid with distinct red blood cells and plasma components. The geometric and structural parameters used in the 1D arterial model were derived from published data. In future work, a patient-specific arterial model incorporating geometry and elasticity measurements could be developed, with its predictions validated against non-invasive clinical data. The selection of the patients utilized in the present study was made after anatomical control, so that our sample is morphologically comparable. In the comparative results of FEVAR, chEVAR, and BEVAR, the different preoperative anatomical characteristics should be considered. To validate the hemodynamic predictions, we could impose patient-specific boundary conditions on each individual patient. A broader investigation involving a larger patient cohort could provide deeper insight into the hemodynamic behavior of individuals undergoing complex endovascular repair. Moreover, incorporating patient-specific boundary conditions for each case would likely enhance the accuracy of the hemodynamic predictions.

## 5. Conclusions

In this study, a multiscale modeling framework was employed, coupling low-order computational models with three-dimensional CFD simulations to investigate postoperative aortic hemodynamics. Key flow-related quantities, including velocity, local normalized helicity (LNH), wall shear stress (WSS), and time-averaged wall shear stress (TAWSS), were analyzed in patient-specific aortic geometries following implantation of advanced endovascular devices, namely BEVAR, chEVAR, and FEVAR.

All three endovascular designs led to an overall improvement in postoperative blood flow characteristics toward near-physiological conditions, with a reduction in adverse flow patterns and recirculation zones. FEVAR demonstrated more stable visceral artery perfusion, and BEVAR exhibited higher flow rates with fewer recirculation regions, while chEVAR showed more streamlined visceral artery flow accompanied by reduced regurgitation at the bridging stent entries. Despite these differences, all configurations effectively preserved visceral artery perfusion.

Overall, all EVAR designs produced comparable hemodynamic outcomes across most parameters and consistently improved flow conditions relative to the pathological state. FEVAR was associated with lower velocity magnitudes and more uniform, smoother helicity distributions compared to BEVAR and chEVAR. In contrast, the multiscale model predicted higher visceral artery flow values in the BEVAR configuration relative to FEVAR. The use of three-dimensional, patient-specific aortic models enhances the clinical relevance of the present findings and supports their potential application in personalized treatment planning. These results may further contribute to the future technological refinement of branch endografts tailored for use in F/BEVAR and chEVAR procedures.

## Figures and Tables

**Table 1:** Main body and bridging stent details of each patient.

Cases	Main Body	Max Aortic Diameter at Proximal Landing Zone	Max Visceral Aortic Diameter	Infrarenal Aortic Angulation	Neck Calcification/Thrombus in >25% of the Circumference of Landing Zone	Bridging Stent	Diameter of TVs at Landing Zone	Angles of TVs
FEVAR								
1	Cook Fenestrated graft: 36 × 22 × 162 mm	30.2 mm	33.0 mm	20.0°	No	RRA: BeGraft 6 × 28 mm LRA: BeGraft 6 × 28 mm SMA: BeGraft 8 × 37 mm CA: BeGraft 9 × 37 mm	RRA: 6.1 mm LRA: 6.1 mm SMA: 7.5 mm	RRA: 25° LRA: 3° SMA: 27°
2	Cook Fenestrated graft: 30 × 22 × 114 mm	26.0 mm	29.6 mm	8.0°	No	RRA: BeGraft 6 × 28 mm LRA: BeGraft 6 × 28 mm SMA: BeGraft 8 × 37 mm	RRA: 6.0 mm LRA: 5.7 mm SMA: 7.6 mm	RRA: 23° LRA: 18° SMA: 10°
3	Cook Fenestrated graft: 38 × 35 × 162 mm	31.5 mm	30.7 mm	17.0°	No	RRA: BeGraft 6 × 28 mm LRA: Begraft 6 × 28 mm SMA: Begraft 8 × 37 mm CA: Begraft 9 × 37 mm	RRA: 5.7 mm LRA: 6.0 mm SMA: 7.7 mm	RRA: 27° LRA: 17° SMA: 23°
ChEVAR								
1	Endurant IIs: 36 × 14 × 103 mm	27.6 mm	32.0 mm	15.8°	No	RRA: BeGraft 6 × 58 mm LRA: BeGraft 6 × 58 mm SMA: BeGraft 8 × 57 mm	RRA: 5.3 mm LRA: 6.0 mm SMA: 7.9 mm	RRA: 32° LRA: 12° SMA: 31°
2	Endurant IIs: 36 × 14 × 103 mm	27.1 mm	27.6 mm	31.0°	No	RRA: Begraft 6 × 58 mm LRA: BeGraft 6 × 58 mm SMA: BeGraft 8 × 57 mm	RRA: 5.1 mm LRA: 5.6 mm SMA: 7.9 mm	RRA: 37° LRA: 8° SMA: 26°
3	Endurant IIs GRAFT: 36 × 14 × 103 mm	27.0 mm	29.0 mm	28.0°	No	RRA: BeGraft 6 × 58 mm LRA: BeGraft 6 × 58 mm SMA: BeGraft 8 × 57 mm	RRA: 5.5 mm LRA: 5.9 mm SMA: 7.7 mm	RRA: 24° LRA: 28° SMA: 43°
BEVAR								
1	Cook t-Branch: 34 × 19 × 202 mm	30.5 mm	36.8 mm	15°	No	RRA: BeGraft 6 × 57 mm LRA: BeGraft 6 × 57 mm CA: BeGraft 8 × 57 mm SMA: BeGraft 8 × 57 mm	RRA: 5.6 mm LRA:5.2 mm CA: 8.1 mm SMA: 7.8 mm	RRA:21° LRA:20° SMA: 17°
2	Cook thoracic graft 49 × 36 × 217 mm+ Cook t-Branch: 34 × 19 × 202 mm	26 mm	32.4 mm	14°	No	RRA: BeGraft 7 × 57 mm LRA: BeGraft 7 × 57 mm CA: BeGraft 9 × 59 mm SMA: BeGraft 8 × 57 mm	RRA: 6.7 mm LRA:7 mm CA: 8.6 mm SMA: 8.1 mm	RRA:6° LRA:16° SMA: 6°
3	Cook t-Branch: 34 × 19 × 202 mm	31 mm	33 mm	15°	No	RRA: BeGraft 6 × 57 mm LRA: BeGraft 7 × 57 mm CA: BeGraft 8 × 59 mm SMA: BeGraft 9 × 57 mm	RRA: 6.5 mm LRA:7.1 mm CA: 8 mm SMA: 8.6 mm	RRA:10° LRA:14° SMA: 101°

**Table 2:** Mean values for the hemodynamic parameters between FEVAR, chEVAR, and BEVAR cohorts.

	**FEVAR**	**ChEVAR**	**BEVAR**	**FEVAR**	**ChEVAR**	**BEVAR**	**FEVAR**	**ChEVAR**	**BEVAR**
**RRT-Mean (1/Pa)**	**SMA**	**SMA**	**SMA**	**RRA**	**RRA**	**RRA**	**LRA**	**LRA**	**LRA**
**P1**	1.0069	0.5225	0.5350	1.0478	1.0941	0.5196	1.4802	0.9316	0.8077
**P2**	0.6430	1.7459	0.9139	0.6088	2.0717	0.7050	0.5181	0.8775	0.6557
**P3**	0.5212	0.8302	0.6503	0.8630	1.0577	0.4820	0.7628	0.9322	0.9160
**Average**	0.7237	1.0328	0.6997	0.8399	1.4078	0.5689	0.9204	0.9138	0.7931
**OSI-Mean (Dimensionless)**	**SMA**	**SMA**	**SMA**	**RRA**	**RRA**	**RRA**	**LRA**	**LRA**	**LRA**
**P1**	0.0070	0.0084	0.0062	0.0061	0.0175	0.0067	0.0107	0.0073	0.0091
**P2**	0.0038	0.0118	0.0095	0.0052	0.0129	0.0064	0.0050	0.0210	0.0045
**P3**	0.0040	0.0073	0.0075	0.0033	0.0053	0.0098	0.0038	0.0057	0.0111
**Average**	0.0049	0.0092	0.0077	0.0048	0.0119	0.0076	0.0065	0.0114	0.0083
**TAWSS-Mean (Pa)**	**SMA**	**SMA**	**SMA**	**RRA**	**RRA**	**RRA**	**LRA**	**LRA**	**LRA**
**P1**	2.0107	1.6635	3.3955	1.8828	0.9943	4.5400	1.7417	1.4609	2.8088
**P2**	2.4245	1.1021	2.5005	2.7818	1.3462	3.0023	2.8822	2.1734	2.1953
**P3**	2.6638	1.9726	3.0866	2.2412	2.7191	4.3557	2.4512	2.1275	2.1453
**Average**	2.3663	1.5794	2.9942	2.3019	1.6865	3.9660	2.3584	1.9206	2.3831
**Mean Flow Rate of the cardiac cycle (l/s)**	**SMA**	**SMA**	**SMA**	**RRA**	**RRA**	**RRA**	**LRA**	**LRA**	**LRA**
**P1**	1.20 × 10^−5^	7.05 × 10^−6^	1.25 × 10^−5^	7.77 × 10^−6^	3.22 × 10^−6^	8.78 × 10^−6^	7.99 × 10^−6^	4.79 × 10^−6^	8.78 × 10^−6^
**P2**	4.53 × 10^−6^	4.39 × 10^−6^	8.67 × 10^−6^	5.77 × 10^−6^	1.59 × 10^−6^	6.88 × 10^−6^	4.78 × 10^−6^	4.14 × 10^−6^	4.14 × 10^−6^
**P3**	8.29 × 10^−6^	3.92 × 10^−6^	1.13 × 10^−5^	5.04 × 10^−6^	1.62 × 10^−6^	6.65 × 10^−6^	5.90 × 10^−6^	2.51 × 10^−6^	5.90 × 10^−6^
**Average**	8.28 × 10^−6^	5.12 × 10^−6^	1.08 × 10^−5^	6.20 × 10^−6^	2.15 × 10^−6^	7.44 × 10^−6^	6.22 × 10^−6^	3.81 × 10^−6^	6.27 × 10^−6^
**Pressure-MAP (Mean Arterial Pressure) (Pa)**	**SMA**	**SMA**	**SMA**	**RRA**	**RRA**	**RRA**	**LRA**	**LRA**	**LRA**
**P1**	12,448.0	12,595.4	13,042.2	12,423.6	12,607.7	12,881.4	12,436.0	12,523.1	12,937.9
**P2**	12,449.0	12,528.7	12,794.0	12,425.3	12,515.9	12,691.8	12,436.3	12,573.5	12,652.1
**P3**	12,447.6	12,447.3	12,903.6	12,423.6	12,423.3	12,889.4	12,436.0	12,437.0	12,986.0
**Average**	12,448.2	12,523.8	12,913.2	12,424.2	12,515.6	12,820.9	12,436.1	12,511.2	12,858.7
**WSS-Mean at peak systole (Pa)**	**SMA**	**SMA**	**SMA**	**RRA**	**RRA**	**RRA**	**LRA**	**LRA**	**LRA**
**P1**	15.75	11.40	23.92	12.61	7.96	31.86	13.09	9.94	26.29
**P2**	21.32	14.95	22.98	20.66	12.23	21.89	22.79	15.52	19.16
**P3**	20.58	27.57	26.27	18.05	19.52	33.24	19.60	18.70	26.67
**Average**	19.22	17.98	24.39	17.11	13.24	28.99	18.50	14.72	24.04

**Table 3:** Percentage of flow reversal in the visceral arteries of FEVAR, chEVAR, and BEVAR cases.

**FEVAR cases**				
**Target vessel**	**Patient 1 (%)**	**Patient 2 (%)**	**Patient 3 (%)**	**Mean Value**
**SMA**	11.1834	24.7713	14.3677	16.7741
**LRA**	5.4558	19.0680	6.2420	10.2552
**RRA**	1.6034	10.3179	6.0401	5.9871
**ChEVAR cases**				
**Target vessel**	**Patient 1 (%)**	**Patient 2 (%)**	**Patient 3 (%)**	**Mean Value**
**SMA**	14.3160	29.9458	20.5075	21.5897
**LRA**	11.9259	21.2901	29.2485	20.8215
**RRA**	10.0024	36.8587	26.1691	24.3434
**BEVAR cases**				
**Target vessel**	**Patient 1 (%)**	**Patient 2 (%)**	**Patient 3 (%)**	**Mean Value**
**SMA**	3.6570	13.2076	12.8335	9.8994
**LRA**	2.2868	23.7582	60.3961	28.0514
**RRA**	2.2868	2.4989	10.3019	5.0292

SMA: superior mesenteric artery; LRA: left renal artery; RRA: right renal artery.

## Data Availability

The original contributions presented in this study are included in the article. Further inquiries can be directed to the corresponding author.

## Abbreviations

The following abbreviations are used in this manuscript:

FEVAR	Fenestrated endovascular repair
BEVAR	Branched endovascular repair
chEVAR	Chimney endovascular repair
CFD	Computational fluid dynamics
WSS	Wall shear stress
AAA	Abdominal aortic aneurysm
RRA	Right renal artery
LRA	Left renal artery
SMA	Superior mesenteric Artery
CA	Celiac artery
RRT	Relative residence time
OSI	Oscillatory shear index
TAWSS	Time-averaged wall shear stress
LNH	Local normalized helicity
